# A real-life illusion of assimilation in the human face: eye size illusion caused by eyebrows and eye shadow

**DOI:** 10.3389/fnhum.2015.00139

**Published:** 2015-03-20

**Authors:** Kazunori Morikawa, Soyogu Matsushita, Akitoshi Tomita, Haruna Yamanami

**Affiliations:** ^1^School of Human Sciences, Osaka UniversitySuita-shi, Japan; ^2^Shiseido Research CenterYokohama, Japan

**Keywords:** illusion, Delboeuf illusion, eye, eyebrow, face, assimilation, eye shadow

## Abstract

Does an assimilative illusion like the Delboeuf illusion occur in the human face? We investigated factors that might influence the perceived size of the eyes in a realistic face. Experiment 1 manipulated the position of the eyebrows (high or low), the presence/absence of eye shadow, and the viewing distance (0.6 m or 5 m), then measured the perceived eye size using a psychophysical method. The results showed that low eyebrows (i.e., closer to the eyes) make the eyes appear larger, suggesting that the assimilation of eyes into the eyebrows is stronger when the eye-eyebrow distance is shorter. The results also demonstrated that the application of eye shadow also makes the eyes look larger. Moreover, the effect of eye shadow is more pronounced when viewed from a distance. In order to investigate the mechanism of the eye size illusion demonstrated in Experiment 1, Experiment 2 measured the magnitude of the Delboeuf illusion at a viewing distance of 0.6 m or 5 m, with or without gray gradation simulating the eye shadow that was used in Experiment 1. The experiment demonstrated that the Delboeuf illusion is modulated by viewing distance and gradation in the same way as the eye size illusion. These results suggest that the eye size illusion induced by the eyebrows and the Delboeuf illusion involve the same mechanism, and that eye shadow causes the assimilation of the eyes into itself and enhances assimilation between the eyes and the eyebrows.

## Introduction

One of the relatively unexplored directions of research on perceptual illusions is the study of illusions in real life. Historically, most visual illusions have been studied with very little relevance to everyday life. Most visual illusion figures have been highly contrived and unnatural stimuli. In a sense, it is not surprising that these artificial stimuli cause unnatural perceptions called illusions because the biological evolution of the visual system did not anticipate such stimuli. However, stimuli used in research on illusions do not have to be so unnatural. Natural illusions, albeit much less dramatic, are far more prevalent in our daily lives than we may think. In fact, some illusions can be very relevant to everyday life (Morikawa, 2003, 2012, 2015). In the present study, we investigate a visual geometric illusion in the most natural and socially important stimulus, that is, the human face.

The eyebrows might influence the perceived size of the eyes because the human visual system perceives and recognizes faces through holistic processing that integrates facial features and their spatial relationships into a global representation of the face (e.g., McKone and Robbins, 2011). In holistic processing, facial features are *interdependent* in the sense that alteration of some features affects the processing of other features (e.g., Young et al., 1987; Tanaka and Sengco, 1997). This raises the question as to whether changing the position of the eyebrows alters the perception of the eyes.

Although the eyebrows constitute one of the most salient features of the human face (Haig, 1985; Nagai et al., 2013), very few previous studies have investigated the perceptual effects of the eyebrows. However, there are at least three reasons as to why the often-neglected eyebrows merit further scientific investigation.

First, eyebrows have a significant impact on face identification. For example, information in the eye and eyebrow regions of facial stimuli was most clearly linked to observers’ ability to discriminate those faces (Gosselin and Schyns, 2001; Sekuler et al., 2004; Nagai et al., 2013). Furthermore, Sadr et al. (2003) demonstrated that it was more difficult to identify faces without eyebrows than to identify faces without eyes. The importance of eyebrows is also indicated by the fact that no other feature in the face can be as easily and dramatically altered as eyebrows by means of cosmetics. Altering eyebrows can cause a change in the overall facial impression (Morikawa, 2012, 2015).

Second, although many make-up artists claim that changing the position and shape of the eyebrows can alter the perceived eye size, this claim has not yet been substantiated. Eyes are an important determinant of facial attractiveness. For example, Baudouin and Tiberghien (2004) showed that wider eyes make female faces more attractive (also Geldart et al., 1999). Gründl et al. (2008) showed that young observers regarded the oblique type of eye axis (higher lateral canthus) as more attractive than the horizontal type. If it is experimentally proven that the position of the eyebrows affects the perceived eye size, there could be practical implications.

Third, geometric illusions arising from the perceptual integration of the eye and eyebrow may explain alterations of eye size perception as a function of eyebrow position. Studying the effects of the eyebrows may uncover naturally occurring geometric illusions in the human face and shed light on the relationship between face perception and visual illusions. Interactions between contours usually manifest themselves as illusions of either assimilation or contrast. In illusions of assimilation, differences in size, orientation, and so forth between the central and surrounding parts of the stimuli appear smaller than they really are. In the Delboeuf illusion, for example, the separation between the inner and outer circles appears lessened when the outer circle is not too large. On the contrary, in illusions of contrast, differences in size, orientation, and so forth between the central and surrounding parts of the stimuli appear exaggerated. In the Ebbinghaus illusion, for example, a circle surrounded by several larger circles seems to shrink (an effect of size contrast). It is possible that such geometric illusions also occur during the perception of faces (Schwaninger et al., 2003; Morikawa, 2012, 2015; Xiao et al., 2014). Eyebrows can be an excellent tool with which to study visual illusions in human faces.

Another factor that may induce an assimilative illusion of eye size is eye shadow, which is a colored cosmetic product applied to the eyelids or the skin around the eyes. The effects of makeup on the perceived shape of the face have seldom been scientifically investigated. Although one of the purposes of eye shadow is to enhance the depth of eyes (Abe et al., 2009), there may be more to eye shadow than just adding depth. Typical eye shadow is darkest along the sharp boundary of the upper eyelid and gradates to the skin tone as it approaches the eyebrow. Thus, it is possible that the eye is assimilated with eye shadow, which may induce an overestimation of the eye size. Another possibility is that eye shadow enhances the perceptual grouping of the eye and the eyebrow through contrast reduction or by the process of color spreading in a manner similar to the water color illusion (Pinna et al., 2003), which may cause the eye to appear larger. If eye shadow indeed has such effects, then it could be another example of a real-life illusion of assimilation.

A natural variable that might modulate the assimilation induced by eyebrows and eye shadow is the viewing distance. As the viewing distance increases, the absolute retinal distance (but not the relative retinal distance) between the eye, eye shadow, and the eyebrow decreases. Generally speaking, perceptual grouping is stronger between parts that are closer to each other on the retina (Masin, 2002). Moreover, van der Kooij and te Pas (2009) and Mareschal et al. (2010) found that adding noise to the stimuli results in increased assimilation. Masin (2002) suggested that, as visibility declines, information is pooled over increasingly large regions of the visual field. Increasing the viewing distance makes the visibility of the eye-eyebrow area somewhat lower by increasing noise, which may result in stronger assimilation of the eye, eye shadow, and the eyebrow. Although the size of the retinal image can be decreased by reducing the stimulus size on the display monitor, this method would decrease the image resolution and change the appearance of the stimulus face. Therefore, we decided to increase the viewing distance instead. From the viewpoint of neurons in the primary visual cortex, a small stimulus viewed from a short distance is equivalent to a large stimulus viewed from a long distance. In real life, however, viewing distance is an important aspect of object perception. Thus, changing the viewing distance is a natural and meaningful manipulation of interest, both scientifically and practically.

This study has two aims. First, we examine if the perceived eye size is affected by the assimilative effects of the eyebrows and eye shadow, and if such effects are modulated by the viewing distance. To do so, we employ psychophysical methods to precisely measure the perceived size of the eyes, as influenced by eye shadow and the position of the eyebrows. Second, we investigate if the eye size illusion induced by the eyebrows is based on the same mechanism as that of the Delboeuf illusion.

## Experiment 1

In this experiment, we measured the perceived size of the eyes in facial photographs in which the position of the eyebrows was either high or low, and eye shadow was present or absent. The eyebrow position was changed by shifting the entire eyebrow up or down. If the assimilation of the eye into the eyebrow occurs to the same extent regardless of the distance between the eye and the eyebrow, then the eye should appear larger when the eyebrow position is higher, as if the eyelid is being lifted up by the eyebrow. However, if the power of assimilation is stronger when the eyebrow is closer to the eye, then the eye should appear larger when the eyebrow position is lower.

If the eye is also assimilated into eye shadow, then the eye with eye shadow should appear larger than the eye without eye shadow. Eye shadow also reduces the difference in luminance between the eyelid and the eyebrow, which may enhance the assimilation of the eye into the eyebrow. Either way, the application of eye shadow should make the eye look larger.

In addition, if making the retinal image smaller strengthens the assimilation of the eyes into the eyebrows and/or eye shadow, viewing the image at a greater distance should intensify the eye size illusion.

### Method

#### Participants

Twenty-two undergraduate students (10 males and 12 females) participated. Their visual acuity was measured with the FrACT 3.7 test (Bach, 1996). All had normal or corrected-to-normal visual acuity of 0.7 or better. The participants were compensated for their time with merchandise considered appropriate in value by university standards.

#### Stimuli and Apparatus

The experiment was run on a computer with a program we created using Microsoft Visual Basic 6.0. The stimuli were presented on a 24.1-inch LCD screen (NEC MultiSync LCD-PA241W, screen size 518 × 324 mm, 1920 × 1200 pixels, refresh frequency 60 Hz, CIE chromaticity coordinates of white: *x* = 0.322, *y* = 0.326). Although we did not secure the observing position with any apparatus, the viewing distance remained constant, at approximately 0.6 m or 5 m.

All facial stimuli were generated from a single facial image that was based on an average face of young Japanese female adults; the image was somewhat modified so as to represent typical attractiveness (Matsushita et al., submitted). The dimensions of the stimuli were 660 pixels wide and 900 pixels high (16.53° × 22.03° in visual angle at 0.6 m and 2.04° × 2.78° at 5 m). The dimensions of the face itself were approximately 480 pixels wide (12.18° in visual angle at 0.6 m and 1.48° at 5 m) at the cheekbone level and approximately 810 pixels high (20.01° at 0.6 m and 2.50° at 5 m) from the top of the head to the tip of the chin. The stimuli were color images and the mean luminance of the facial area was 114.1 cd/m^2^. The background of the facial images was slightly colored gray with a median RGB of 214, 219 and 213.

**Figure 1:**
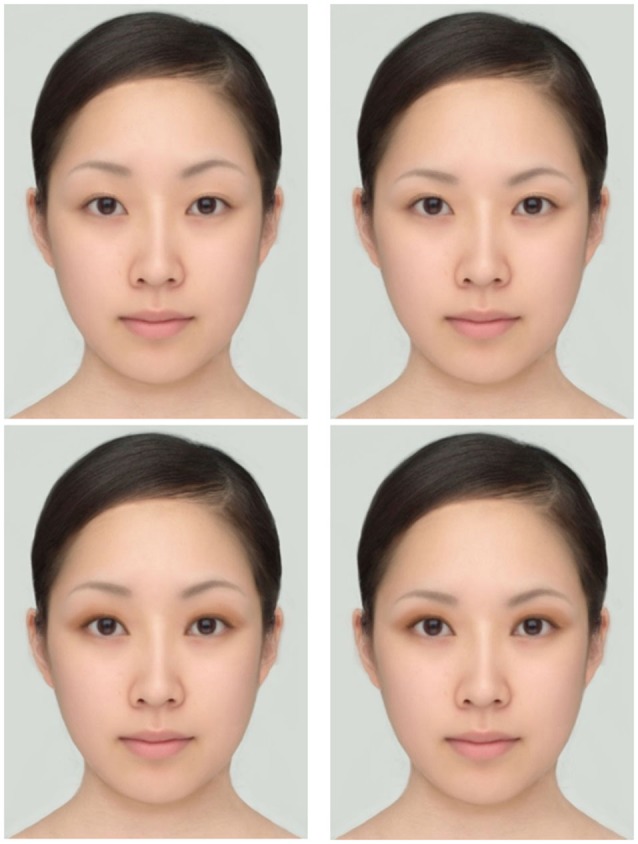
**The standard stimuli used in Experiment 1**. The top row shows the faces without eye shadow, and the bottom row shows the faces with eye shadow. The left column shows the faces with high eyebrows, and the right column shows the faces with low eyebrows. Except for the eyebrow position and eye shadow, the faces are identical.

**Figure 2:**
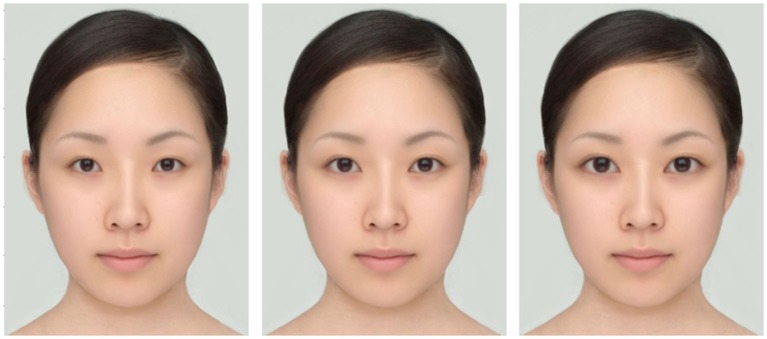
**Samples of the comparative stimuli used in Experiment 1**. Left: 90% eye size. Center: 100% eye size. Right: 110% eye size.

The standard stimuli were facial images with a manipulated eyebrow position (Figure 1). The position of the eyebrows was shifted either 8 pixels downward or 8 pixels upward from the original eyebrow position (Low Condition and High Condition, respectively). In addition, for each level of the eyebrow position, the application of brown eye shadow was simulated using digital photograph editing software PaintShop Pro XI. Hence, the total number of the standard stimuli was four. In the original face, the vertical length of the eye (i.e., the distance between the top and bottom of the palpebral fissure) was 36 pixels and the distance between the top of the eye and the lower edge of the eyebrow was 45 pixels on the vertical line that passed through the center of the pupil. Hence, a shift of 8 pixels was equivalent to 18% of the original eye-eyebrow distance. Comparative stimuli to be used in the staircase method were facial images without eye shadow; the eye size in these stimuli was sequentially changed from 90% to 110% of the original eye size (i.e., 100%) in steps of 2% both horizontally and vertically (Figure 2).

#### Procedure

The experiments were approved by the ethical board of the School of Human Sciences of Osaka University. After the participants signed the informed consent forms and received the instructions, the experimental task started.

During each trial, a standard stimulus and a comparative stimulus were displayed side by side on the computer screen. The background of the images was gray. During the presentation, the participants moved their eyes freely to compare the two faces. Following the presentation of the stimuli for 1500 ms, the screen changed to blank-gray, which lasted for at least 1500 ms and until the participants responded. The participants’ task was to judge which face appeared to have larger eyes. We instructed the participants not to focus on a few specific points of the stimulus, but to pay attention to the whole area of the face. Following the response, the next stimulus pair was presented. To measure the eye size of the comparative stimulus that was perceived to be the same as that of the standard stimulus, we used the staircase method, also known as the up-and-down method. For each standard stimulus, there was one staircase; thus, the experiment consisted of four concurrent staircases of trials, which were randomly interleaved. Whether the standard stimulus was presented on the left or right hand side of the screen was determined randomly on each trial. The eyes of the comparative stimulus for the first trial of each staircase were either obviously smaller (ascending series) or obviously larger (descending series) than those of the standard stimulus. Each staircase was terminated when the direction of the staircase was reversed nine times. The experiment took approximately 20 min.

### Results and Discussion

**Figure 3:**
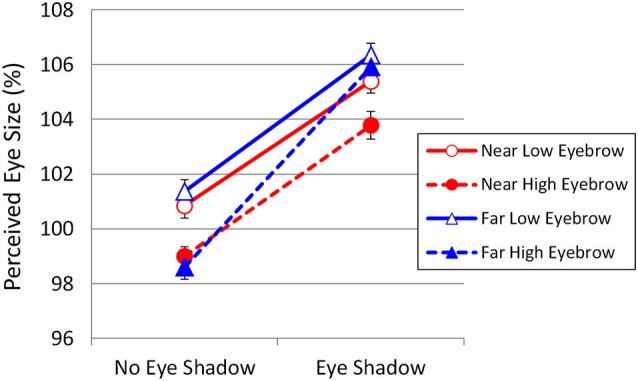
**Results of Experiment 1**. Perceived eye size as a function of eyebrow position, eye shadow, and viewing distance. Vertical bars indicate standard errors.

First, we computed the point of subjective equality (PSE) for each standard stimulus; the PSE was the mean of the last eight eye-size values of the comparative stimuli wherein the staircase direction had been reversed from upward to downward or from downward to upward. The results are shown in Figure 3. The mean perceived eye size without eye shadow was 101.0% and 98.8% for the Low and High eyebrow conditions, respectively. The mean perceived eye size with eye shadow was 105.9% and 104.8% for the Low and High eyebrow conditions, respectively.

A three-way repeated-measures ANOVA with eye shadow, eyebrow position, and viewing distance as factors indicated that the main effect of eye shadow was highly significant, *F*_(1,21)_ = 222.08, *p* < 0.001, $\eta_{p}^{2}$ = 0.914. In addition, the main effects of eyebrow position and viewing distance were significant (*F*_(1,21)_ = 20.55, *p* < 0.001, $\eta_{p}^{2}$ = 0.495 and *F*_(1,21)_ = 9.07, *p* = 0.007, $\eta_{p}^{2}$ = 0.302, respectively). The interaction between eye shadow and viewing distance was significant (*F*_(1,21)_ = 8.19, *p* = 0.009, $\eta_{p}^{2}$ = 0.281), indicating that eyes with eye shadow were perceived as larger at 5 m than at 0.6 m. Moreover, the interaction between eye shadow and eyebrow position was significant (*F*_(1,21)_ = 10.78, *p* = 0.004, $\eta_{p}^{2}$ = 0.339), revealing that, without eye shadow, high eyebrows made the eyes appear significantly smaller than did low eyebrows. However, the interaction between eyebrow position and viewing distance was not significant (*F*_(1,21)_ = 0.35, *p* = 0.85, $\eta_{p}^{2}$ = 0.002). In addition, the three-way interaction was significant (*F*_(1,21)_ = 5.79, *p* = 0.025).

Analysis of simple main effects confirmed that the viewing distance affected the eye size illusion in the presence of eye shadow (*F*_(1,42)_ = 17.261, *p* = 0.0002, $\eta_{p}^{2}$ < 0.291), but not in its absence (*F*_(1,42)_ = 0.028, *p* = 0.867, $\eta_{p}^{2}$ < 0.001). Moreover, analysis of simple-simple main effects revealed that increasing the viewing distance to 5 m in the presence of eye shadow enhanced the overestimation of the eye size only when the eyebrows were high (*F*_(1,84)_ = 14.853, *p* = 0.0002, $\eta_{p}^{2}$ = 0.150), but not when they were low (*F*_(1,84)_ = 2.965, *p* = 0.089, $\eta_{p}^{2}$ = 0.034).

Low eyebrows always made the eyes appear significantly larger than did high eyebrows, except in the condition in which eye shadow had been added, with the stimulus viewed at 5 m. In fact, eye shadow had the greatest effect in the high-eyebrow condition, with the stimulus viewed at 5 m. This result suggests that the addition of eye shadow can overcome the eye-reducing effect of high eyebrows when viewed from a distance, perhaps because eye shadow bridges the gap between the eye and the eyebrow and enhances the assimilation of the eyes into the eyebrows.

**Figure 4:**
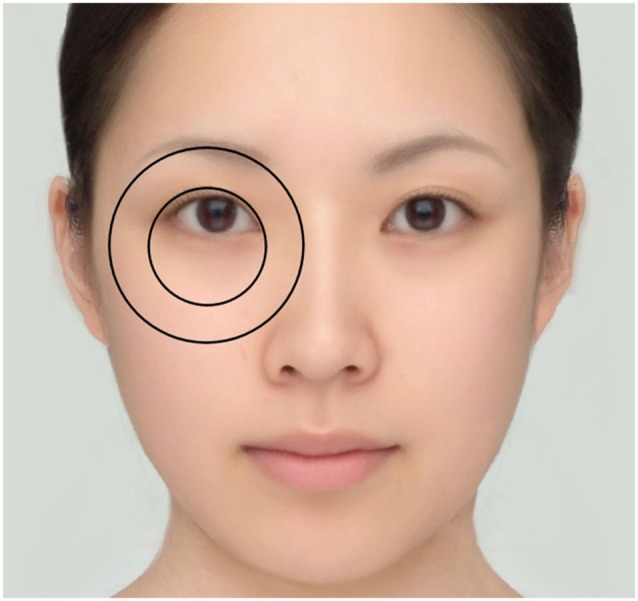
**The hypothetical relationship between the eye size illusion and the Delboeuf illusion**. In this figure, the position of the eyebrows is halfway between the Low and High conditions of Experiment 1. The diameter ratio of the inner circle to the outer circle is 2:3.3, which is halfway between the Small and Large conditions of Experiment 2.

The results did not show an “eyelid-lifting effect” with a high eyebrow. Instead, the eyebrow position had the opposite effect; the perceived eye size was larger when the eyebrow was low. In fact, the high eyebrows resulted in eye size underestimation. *T*-tests indicated that the mean values for the Near High and Far High eyebrows in the No Eye Shadow condition (99.0% and 98.6%, respectively) were significantly smaller than 100% (*t*_(21)_ = 2.939, *p* = 0.008 and *t*_(21)_ = 3.185, *p* = 0.004, respectively). However, this underestimation does not necessarily mean size contrast, because it is likely that even high eyebrows make eyes appear larger than when there are no eyebrows at all. This result indicates that the mere presence of eyebrows, regardless of their position, makes eyes appear larger than they really are and that decreasing the distance between the eye and the eyebrow increases the power of assimilation. The relationship between the eye–eyebrow distance and the strength of assimilation may be analogous to the Delboeuf illusion, which is an illusion of assimilation wherein an inner circle is assimilated to an outer circle and appears larger than it really is (Figure 4).

## Experiment 2

The purpose of Experiment 2 was to examine whether the eye size illusion in Experiment 1 is based on the same mechanism as that of the Delboeuf illusion. We measured the magnitude of the Delboeuf illusion at a viewing distance of 0.6 m or 5 m, in the presence or absence of a gray gradation that simulated the eye shadow used in Experiment 1. If the eye size illusion in Experiment 1 and the Delboeuf illusion indeed involve the same mechanism, then the Delboeuf illusion should be modulated by the viewing distance and gradation in the same way as the eye size illusion is.

### Method

#### Participants

Twenty-two undergraduate students (10 males and 12 females) participated. They also participated in Experiment 1. Their visual acuity was measured with the FrACT 3.7 test (Bach, 1996). All had normal or corrected-to-normal visual acuity of 0.7 or better.

#### Stimuli and Apparatus

**Figure 5:**
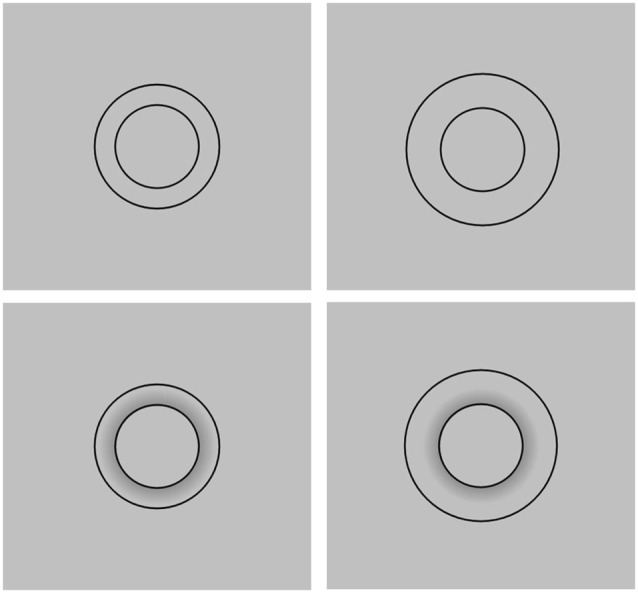
**The standard stimuli used in Experiment 2**. The top row shows the Small (left) and Large (right) conditions with no gradation. The bottom row shows the Small and Large conditions with gray gradation. All the inner circles are identical in size.

The same apparatus as that of Experiment 1 was used. The standard stimuli were concentric circles drawn in a black line on a light-gray background, presented on a computer screen (Figure 5). The diameter of the inner circle was 122 pixels, which subtended a visual angle of 3.14° and 0.38° at a viewing distance of 0.6 m and 5 m, respectively. The diameters of the small outer circle (Small Condition) and the large outer circle (Large Condition) were 182 pixels (4.68° and 0.56° at 0.6 m and 5 m) and 222 pixels (5.70° and 0.69° at 0.6 m and 5 m), respectively. The diameter ratio of the inner circle to the outer circle was 2:3 for the Small Condition and 2:3.64 for the Large Condition. In addition, for each level of the outer circle diameter, an identical gray gradation was digitally added around the inner circle. Thus, the total number of the standard stimuli was four. The gradation simulated the eye shadow used in Experiment 1, and was light enough to be visually distinct from the inner circle.

The comparative stimulus was a single circle with a diameter that was sequentially decreased or increased by 2% according to the observers’ judgment, using the same staircase method as that in Experiment 1. During each trial, a standard stimulus and a comparative stimulus were presented side by side. Whether the standard stimulus was presented on the left or right hand side of the screen was determined randomly on each trial.

#### Procedure

The same procedure as that of Experiment 1 was used, except that the observers judged which, between the inner circle and the single comparative circle, appeared larger.

### Results and Discussion

**Figure 6:**
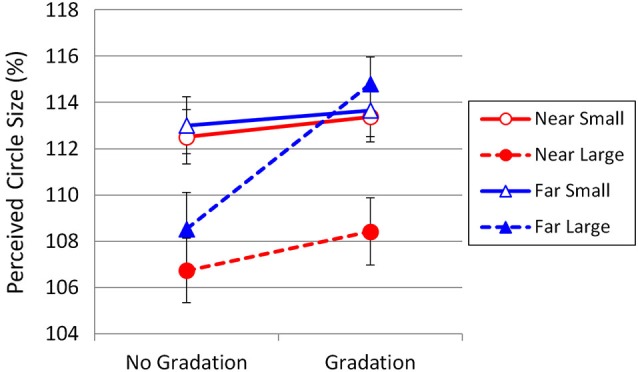
**Results of Experiment 2**. Perceived inner circle size as a function of outer circle size, gradation, and viewing distance. Vertical bars indicate standard errors.

The PSE was calculated in the same way as in Experiment 1. The results are shown in Figure 6. The mean perceived circle size without gradation was 112.8% and 107.6% for the Small and Large conditions, respectively. The mean perceived circle size with gradation was 113.5% and 111.6% for the Small and Large conditions, respectively. Overall, the illusion magnitudes obtained in Experiment 2 were much greater than those in Experiment 1. This is not surprising because the eye and eyebrow correspond to only a fraction of the concentric circles that constitute the Delboeuf illusion figure (Figure 4). Weintraub and Schneck (1986) reported that the more the outer circumference is deleted, then the weaker the Delboeuf illusion becomes.

A three-way repeated-measures ANOVA with gradation, outer circle size, and viewing distance as factors indicated a significant main effect of gradation, *F*_(1,21)_ = 10.35, *p* = 0.004, $\eta_{p}^{2}$ = 0.330. The main effects of outer circle size and viewing distance were also significant (*F*_(1,21)_ = 17.42, *p* < 0.001, $\eta_{p}^{2}$ = 0.453 and *F*_(1,21)_ = 5.07, *p* = 0.035, $\eta_{p}^{2}$ = 0.195, respectively). The interaction between gradation and outer circle size was significant (*F*_(1,21)_ = 14.20, *p* = 0.001, $\eta_{p}^{2}$ = 0.403), indicating that the addition of gradation increased the illusion induced by the large outer circle more than the illusion induced by the small outer circle. In addition, the interaction between the size of the outer circle and the viewing distance was significant (*F*_(1,21)_ = 7.60, *p* = 0.012, $\eta_{p}^{2}$ = 0.266), indicating that an increase in the viewing distance amplified the illusion induced by the large outer circle more than the illusion induced by the small outer circle. The interaction between gradation and the viewing distance was marginally significant (*F*_(1,21)_ = 4.03, *p* = 0.058, $\eta_{p}^{2}$ = 0.161), suggesting that the effectiveness of gradation was somewhat greater at 5 m than at 0.6 m. The three-way interaction was also significant (*F*_(1,21)_ = 11.00, *p* = 0.003, $\eta_{p}^{2}$ = 0.344).

Analysis of simple main effects confirmed that the viewing distance affected the Delboeuf illusion in the presence of gradation (*F*_(1,42)_ = 8.642, *p* = 0.0053, $\eta_{p}^{2}$ < 0.171), but not in its absence (*F*_(1,42)_ = 1.020, *p* = 0.318, $\eta_{p}^{2}$ = 0.0237). Moreover, analysis of simple-simple main effects revealed that increasing the viewing distance to 5 m in the presence of gradation enhanced the overestimation of the inner circle with the large outer circle (*F*_(1,84)_ = 21.829, *p* < 0.0001, $\eta_{p}^{2}$ = 0.206), but not with the small outer circle (*F*_(1,84)_ = 0.042, *p* = 0.838, $\eta_{p}^{2}$ = 0.0005).

Similar to the effects of the low eyebrows in Experiment 1, the small outer circle always made the inner circle appear significantly larger than did the large outer circle, except for the condition in which gradation was viewed at 5 m. In fact, the greatest effect of gradation occurred in the large outer circle condition when viewed at 5 m, just as the greatest effect of eye shadow occurred in the high-eyebrow condition when viewed at 5 m in Experiment 1. This result suggests that the addition of gradation can intensify the weak illusion of the large outer circle when viewed from a distance, perhaps because gradation bridges the gap between the inner and outer circles and enhances assimilation of the inner circle into the outer circle. Thus, the pattern of the results obtained in Experiment 2 closely matches that of Experiment 1.

## General Discussion

In the present study, we used psychophysical methods to investigate whether assimilative illusions occur in the human face. Experiment 1 demonstrated that the perceived eye size is affected by the assimilative effects of the eyebrows and eye shadow, and that such effects are influenced by the viewing distance. The eye appears larger when the eyebrow position is lower, which suggests that the power of assimilation is stronger when the eyebrow is closer to the eye. Eye shadow also makes the eye appear larger. Experiment 2 measured the effects of viewing distance and gradation simulating eye shadow on the Delboeuf illusion, so as to investigate whether the eye size illusion induced by the eyebrows is based on the same mechanism as that of the Delboeuf illusion. The results demonstrated that the Delboeuf illusion is modulated by gradation and viewing distance in the same way as the eye size illusion is, suggesting that the eye size and Delboeuf illusions indeed involve the same mechanism despite the large differences in appearance and illusion magnitude.

Abe et al. (2009) showed that eye shadow increases apparent eye depth and size, although they did not measure the absolute magnitude of the illusion. Abe et al. (2009) argued that an increase in subjective distance between the observer and the eyes leads to an overestimation of eye size due to constancy scaling. However, the magnitudes of the eye size illusion caused by eye shadow in the present study are far too large to be explained by constancy scaling. To induce an overestimation of 5% at a viewing distance of 0.6 m by constancy scaling alone, the eyes would have to be perceived to be 3 cm back; to induce an overestimation of 6% at a viewing distance of 5 m by constancy scaling alone, the eyes would have to be perceived to be 30 cm back in the head. Therefore, eye shadow must cause more effects than just enhancing the depth of eyes, which we believe are an illusion of assimilation.

What is the mechanism of the eye shadow/gradation effect? Does eye shadow make the eye appear larger because the eye is directly assimilated into eye shadow, or because eye shadow enhances the assimilation of the eye into the eyebrow? If the eye shadow effect was based only on the assimilation of the eye into eye shadow, and not in any way related to the eyebrow, then the size of the eye shadow effect would be the same regardless of the eyebrow position. However, this was not the case. Of particular interest is the fact that increasing the viewing distance to 5 m enhances the overestimation of the eye size and the inner circle size only in the high-eyebrow condition with eye shadow and in the condition of the large outer circle with gradation, respectively. The viewing distance has no effect on the illusions when the eyebrow is low, the outer circle is small, or eye shadow/gradation is absent. These results suggest that eye shadow/gradation functions as an assimilative bridge between the two parts (i.e., the eye and eyebrow, or the inner and outer circles), and that the bridge becomes particularly effective when the space between these is relatively large. However, the illusion induced by eye shadow is, on average, about 5%, which is much greater than the approximate 2% illusion induced by the eyebrow position. Therefore, eye shadow may have more functions than just enhancing the assimilation of the eye into the eyebrow. It is likely that the eye shadow/gradation effect involves both the assimilation of the eye/inner circle into eye shadow/gradation and enhancement of the assimilation of the eye/inner circle into the eyebrow/outer circle.

From the viewpoint of assimilation, as opposed to contrast, the effect of eye-eyebrow distance on the perceived size of the eyes could be interpreted as an illusion of contrast in size between the eye (i.e., palpebral fissure) and the area between the upper edge of the eye and the lower edge of the eyebrow. Xiao et al. (2014) reported that eyes embedded in a larger face frame are perceived as smaller than eyes of the same size embedded in a smaller face frame; these authors considered this a size-contrast illusion similar to the Ebbinghaus illusion. In the present study, the high eyebrow leaves a larger space between the eye and the eyebrow than does the low eyebrow. Therefore, it may be possible that the larger space between the eye and the high eyebrow makes the eye appear relatively smaller, whereas the contrast in size is not so pronounced for the smaller space between the eye and the low eyebrow.

The eye size illusion in the present study is more likely to be an illusion of assimilation in which the eye becomes assimilated toward the eyebrow than an illusion of contrast. This speculation is supported by previous research on the Delboeuf illusion. When the diameter ratio of the inner circle to the outer circle is varied in the Delboeuf illusion, the magnitude of the overestimation of the inner circle reaches a maximum at a diameter ratio of 2:3 (Oyama, 1960, 1962; Goto et al., 2007). If the eye and the eyebrow correspond partially to the inner and outer circles of the Delboeuf illusion (Figure 4), then the “diameter ratio” with the low eyebrow is closer to the optimum 2:3 than that with the high eyebrow. Although at diameter ratios of 1:5 or smaller, assimilation gives way to contrast and the inner circle of the Delboeuf illusion figure becomes underestimated, such extreme ratios are not possible in the natural human face. Therefore, the eye size illusion in the present study probably arises from assimilation, not contrast.

Morikawa (2012, 2015) suggested that illusions in the human face and body tend to be in the direction of assimilation, rather than that of contrast. One of the reasons for the predominance of assimilation may be the fact that spaces between facial parts or between body parts are filled with and connected to tissue such as skin, muscles, and bones, unlike the empty spaces between the lines that constitute classical geometric illusions. Moreover, the development of different parts of an individual’s body is often governed and controlled by the same genetic and hormonal mechanisms. Therefore, if an individual’s eyebrows are drooping, his or her eyes are likely to droop, as well. If one part of the body is thin, other parts are also likely to be thin. We hypothesize that eyebrows induce an eye size illusion because the visual system takes these biological co-occurrences and natural correlations into account (Morikawa, 2012, 2015).

Morikawa (2012, 2015) also pointed out that, when geometric illusions in the human face and body are psychophysically measured, the maximum illusion magnitudes tend to be around 5%, a sort of “magic number.” That is exactly what Experiment 1 replicated. An illusion magnitude of 5% is small, as compared to well-known geometric illusions. Perhaps, there might be a natural upper limit to the visual illusions in the human face and body so that the illusions can occur only to the extent that the resulting distortions do not appear unnatural.

The fact that the error bars in Figure 3 are very short indicates that the observers’ perceptual judgments were consistent and stable, even though the magnitudes of the illusions caused by the eyebrow and eye shadow were rather small (about 2% and 5%, respectively). This may be because the human visual system is especially tuned to detecting fine differences in the human face. Identifying and recognizing faces is an extremely important skill in society. We are so sensitive to the configuration of facial features that we can perceive even a very small difference reliably (Morikawa, 2012, 2015).

Our results imply that eyebrows may affect facial attractiveness not only directly, but also indirectly, through their influence on the perceived size of the eyes. People tend to find larger eyes more attractive (Geldart et al., 1999; Baudouin and Tiberghien, 2004). Feser et al. (2007) found that young observers (up to 30 years of age) prefer faces whose eyebrows are in a lower position (i.e., closer to the eyes). Our results might partly explain Feser et al.’s finding; lower eyebrows induce an illusory overestimation of the eye size, which may lead to more attractiveness.

To see if eyebrow position has an effect on the perceived attractiveness of the current stimuli viewed by Japanese students, we conducted an additional survey presenting the face with high eyebrows (Figure 1 top left) and with low eyebrows (Figure 1 top right) and asking which face appeared more attractive than the other. The two faces were printed side by side on a sheet of paper with the left-right position being counterbalanced. Of the 56 students, 73% chose the face with low eyebrows as the more attractive. This result replicated the finding of Feser et al. (2007). Therefore, eyebrow position does influence perceived attractiveness, possibly by making the eyes appear larger.

The current findings regarding the shared mechanism underlying the eye size illusion and the Delboeuf illusion should be interpreted with caution because of a few limitations of this study. First, the differing magnitudes of the illusions make the comparison between Experiments 1 and 2 somewhat problematic. Ideally, the magnitude of the Delboeuf illusion should be somehow rendered approximately equal to that of the eye size illusion. Second, it would be more convincing to show that stimulus manipulations that weaken the Delboeuf illusion also decrease the eye size illusion. Such a shared “negative” result would help in distinguishing the assimilative effect seen in the eye size illusion from a more general effect of context/contrast.

Our findings suggest that researchers should exercise caution when they use cut-out, isolated facial features (e.g., the eyes, nose, and mouth on their own) as experimental stimuli in studies on face perception. This is because isolated facial features may be perceived somewhat differently when they are embedded in a whole face, due to visual illusions caused by neighboring features. Thus, identical facial features may appear different, depending on whether they are shown in isolation or as part of the whole face.

The present study further suggests that one of the mechanisms by which cosmetics and make-up alter facial appearances is to induce visual illusions in the face. Our results demonstrated that cosmetic illusions can be quantitatively measured using psychophysical methods. We believe that measurement and analysis of cosmetic illusions will become a new and fruitful field of psychophysical research in the future.

## Conflict of Interest Statement

The authors declare that the research was conducted in the absence of any commercial or financial relationships that could be construed as a potential conflict of interest.

## References

[B1] Abe T. Sato C. Endo M. (2009). Effect of eye shadow on eye size perception: an experimental examination manipulating the position, area and darkness of eye shadow. J. Jpn. Acad. Facial Stud. 9, 111–118.

[B2] Bach M. (1996). The Freiburg visual acuity test—automatic measurement of visual acuity. Optom. Vis. Sci. 73, 49–53. 10.1097/00006324-199601000-00008 8867682

[B3] Baudouin J. Y. Tiberghien G. (2004). Symmetry, averageness and feature size in the facial attractiveness of women. Acta Psychol. (Amst) 117, 313–332. 10.1016/j.actpsy.2004.07.002 15500809

[B4] Feser D. K. Gründl M. Eisenmann-Klein M. Prantl L. (2007). Attractiveness of eyebrow position and shape in females depends on the age of the beholder. Aesthetic Plast. Surg. 31, 154–160. 10.1007/s00266-006-0149-x 17235461

[B5] Geldart S. Maurer D. Carney K. (1999). Effects of eye size on adults’ aesthetic ratings of faces and 5-month-olds’ looking times. Perception 28, 361–374. 10.1068/p2885 10615473

[B6] Gosselin F. Schyns P. G. (2001). Bubbles: a technique to reveal the use of information in recognition tasks. Vision Res. 41, 2261–2271. 10.1016/s0042-6989(01)00097-9 11448718

[B7] Goto T. Uchiyama I. Imai A. Takahashi S. Hanari T. Nakamura S. (2007). Assimilation and contrast in optical illusions. Jpn. Psychol. Res. 49, 33–44 10.1111/j.1468-5884.2007.00330.x

[B8] Gründl M. Klein S. Horczakiwskyj R. Feser D. Jung M. Eisenmann-Klein M.. (2008). The “jaguar’s eye” as a new beauty trend? Age-related effects in judging the attractiveness of the oblique eye axis. Aesthetic Plast. Surg. 32, 915–919. 10.1007/s00266-008-9180-4 18506510

[B9] Haig N. D. (1985). How faces differ – a new comparative technique. Perception 14, 601–615. 10.1068/p140601 3836392

[B10] Mareschal I. Morgan M. J. Solomon J. A. (2010). Cortical distance determines whether flankers cause crowding or the tilt illusion. J. Vis. 10:13. 10.1167/10.8.13 20884588

[B11] Masin S. C. (2002). Absolute and relative effects of similarity and distance on grouping. Perception 31, 799–811. 10.1068/p3227 12206528

[B12] McKone E. Robbins R. (2011). “Are faces special?,” in The Oxford Handbook of Face Perception, eds Calder A. J. Rhodes G. Johnson M. H. Haxby J. V. (New York: Oxford University Press), 149–176.

[B13] Morikawa K. (2003). An application of the Müller-Lyer illusion. Perception 32, 121–123. 10.1068/p3437 12613791

[B14] Morikawa K. (2012). New directions in research on visual illusions of shape and size related to the human face and body: illusions caused by makeup and clothing. Jpn. Psychol. Rev. 55, 348–361.

[B15] Morikawa K. (2015). “Geometric illusions in the human face and body,” in Oxford Compendium of Visual Illusions, eds Shaprio A. Todorovic D. (New York: Oxford University Press).

[B16] Nagai M. Bennett P. J. Rutherford M. D. Gaspar C. M. Kumada T. Sekuler A. B. (2013). Comparing face processing strategies between typically-developed observers and observers with autism using sub-sampled-pixels presentation in response classification technique. Vision Res. 79, 27–35. 10.1016/j.visres.2013.01.001 23321026

[B17] Oyama T. (1960). Japanese studies on the so-called geometrical-optical illusions. Psychologia 3, 7–20.

[B18] Oyama T. (1962). The effect of hue and brightness on the size-illusion of concentric circles. Am. J. Psychol. 75, 45–55. 10.2307/1419541 14482946

[B19] Pinna B. Werner J. S. Spillmann L. (2003). The watercolor effect: a new principle of grouping and figure-ground organization. Vision Res. 43, 43–52. 10.1016/s0042-6989(02)00132-3 12505603

[B20] Sadr J. Jarudi I. Sinha P. (2003). The role of eyebrows in face recognition. Perception 32, 285–293. 10.1068/p5027 12729380

[B21] Schwaninger A. Ryf S. Hofer F. (2003). Configural information is processed differently in perception and recognition of faces. Vision Res. 43, 1501–1505. 10.1016/s0042-6989(03)00171-8 12782063

[B22] Sekuler A. B. Gaspar C. M. Gold J. M. Bennett P. J. (2004). Inversion leads to quantitative, not qualitative, changes in face processing. Curr. Biol. 14, 391–396. 10.1016/j.cub.2004.02.028 15028214

[B23] Tanaka J. W. Sengco J. A. (1997). Features and their configuration in face recognition. Mem. Cognit. 25, 583–592. 10.3758/bf03211301 9337578

[B24] van der Kooij K. te Pas S. F. (2009). Uncertainty reveals surround modulation of shape. J. Vis. 9:15. 10.1167/9.3.15 19757954

[B25] Weintraub D. J. Schneck M. K. (1986). Fragments of Delboeuf and Ebbinghaus illusions: contour/context explorations of misjudged circle size. Percept. Psychophys. 40, 147–158. 10.3758/bf03203010 3774497

[B26] Xiao W. S. Fu G. Quinn P. C. Sun Y.-H. Xiao N. Wang Q.. (2014). The eye-size illusion: psychophysical characteristics, generality and relation to holistic face processing. Perception 43, 265–274. 10.1068/p7647 25109017

[B27] Young A. W. Hellawell D. Hay D. C. (1987). Configurational information in face perception. Perception 16, 747–759. 10.1068/p160747 3454432

